# Neurosurgical fellowship in Europe: It's time to cooperate – A call from the EANS Young Neurosurgeons' Committee

**DOI:** 10.1016/j.bas.2023.102734

**Published:** 2023-12-14

**Authors:** Cesare Zoia, Giorgio Mantovani, Cristina Aldea, Jiri Bartek, Marlies Bauer, Diogo Belo, Evangelos Drosos, Stanislav Kaprovoy, Felix Stengel, Milan Lepic, Laura Lippa, Malte Mohme, Stefan Motov, Michael Schwake, Toma Spiriev, Fabio Torregrossa, Claudius Thomé, Torstein R. Meling, Giovanni Raffa

**Affiliations:** Neurosurgery Unit, Ospedale Moriggia Pelascini, Gravedona, Italy; Neurosurgery Unit, Department of Translational Medicine, University of Ferrara, Ferrara, Italy; National Institute of Neurosciences, Budapest, Hungary; Karolinska University Hospital, Stockholm, Sweden; Department of Neurosurgery, Medical University Innsbruck, Innsbruck, Austria; Neurosurgery Department, Centro Hospitalar Lisboa Norte (CHLN), Lisbon, Portugal; Manchester Centre for Clinical Neurosciences, Manchester, UK; Burdenko Neurosurgical Center, Department of Spinal and Peripheral Nerve Surgery, Department of International Affairs, Moscow, Russia; Kantonsspital, St. Gallen, Switzerland; Military Medical Academy, Belgrade, Serbia; Department of Neurosurgery, Grande Ospedale Metropolitano Niguarda, Milan, Italy; Department of Neurosurgery, University Medical Center Hamburg-Eppendorf, Hamburg, Germany; Kantonsspital, St. Gallen, Switzerland; University Hospital Münster, Germany; Acibadem CityClinic University Hospital Tokuda, Sofia, Bulgaria; Neurosurgical Unit, University of Palermo, Palermo, Italy; Department of Neurosurgery, Medical University Innsbruck, Innsbruck, Austria; Department of Neurosurgery, The National Hospital, Rigshospitalet, Copenhagen, Denmark; Division of Neurosurgery, BIOMORF Department, University of Messina, Italy

To the Editor,

Neurosurgical training across Europe has undergone significant changes in the last 20 years, and even more profound revolutions are yet to come (Stienen et al., 2019, 2020; Zoia et al., 2022; Stengel et al., 2022; Bongetta and Zoia, 2022). Several factors have contributed to this shift, including a decreasing case-load per trainee, working time regulations, and higher quality standards and requirements, among others. It is time to transition from time-based training to competency-based training, as previously discussed in detail (Whitfield et al., 2023; Moiraghi et al., 2020; Perin et al., 2021a, 2021b, 2023; Meling and Meling, 2021). Moreover, in recent years we also witnessed an increasing request of neurosurgeons for expertise in a specific subspecialty.

In this dynamic and evolving scenario, clinical and research fellowships represent a fundamental opportunity for every neurosurgical resident or young neurosurgeon eager to refine their surgical skills, acquire new technical and clinical competencies, and contribute to research and academic productivity. Given the growing specialization within the realm of neurosurgery, fellowships provide aspiring professionals with a dedicated time to enhance their skills and refine their expertise in their subspecialty of choice. In addition, spending time abroad in a structured fellowship offers the opportunity to explore different perspectives, build a robust professional network, and disseminate new ideas and concepts to further improve the overall quality of the training period. In fact, although quantitative data are lacking, there is a general perception that the number of trainees seeking fellowships has steadily increased over the last few years (Clark et al., 2014).

However, the European fellowship landscape is complex and diverse. Currently, a resident or a young neurosurgeon willing to participate must navigate a series of academic, administrative, legal, and logistical challenges, which often hinder them from realizing their projects. Each country has its unique and separate regulations, and fellowship opportunities are scattered, with some old positions still open and new ones unknown to the majority of interested individuals (Niemela and Hernesniemi, 2014).

A dual improvement is strongly needed to offer the best and most streamlined fellowship options to as many trainees as possible. On the one hand, there should be a formal simplification of the laws governing the movement of healthcare workers across Europe at least during training. While it would be a complex process, the European Parliament has already shown interest in our training and working practices. On the other hand, there is a need for the implementation of an already existing common and up-to-date database of fellowship opportunities, with each project sharing its requirements, tasks, objectives, and outcome assessments. In this regard, we can look to our U.S. counterparts, where a similar approach has been adopted by the AANS over the last 20 years, resulting in incredibly positive outcomes.

We strongly advocate the establishment of a European Fellowship program to provide new educational opportunities to young neurosurgeons. A collective and well-directed effort by the EANS in all its Sections and National Societies is the key to providing the next generations of neurosurgeons with a truly European-based fellowship program of unprecedented quality that encompasses all three pillars of the EANS: Training, Education, and Research.

## Declaration of competing interest

The authors have no competing interests to declare that are relevant to the content of this article.
